# 693. Performance of ICD Code Versus Discharge Summary based Query for Endocarditis Cohort Identification

**DOI:** 10.1093/ofid/ofab466.890

**Published:** 2021-12-04

**Authors:** H Nina Kim, Ayushi Gupta, Kristine F Lan, Jenell C Stewart, Shireesha Dhanireddy, Maria A Corcorran

**Affiliations:** University of Washington, Seattle, WA

## Abstract

**Background:**

Studies on infective endocarditis (IE) have relied on International Classification of Diseases (ICD) codes to identify cases but few have validated this method which may be prone to misclassification. Examination of clinical narrative data could offer greater accuracy and richness.

**Methods:**

We evaluated two algorithms for IE identification from 7/1/2015 to 7/31/2019: (1) a standard query of ICD codes for IE (ICD-9: 424.9, 424.91, 424.99, 421.0, 421.1, 421.9, 112.81, 036.42 and ICD-10: I38, I39, I33, I33.9, B37.6 and A39.51) with or without procedure codes for echocardiogram (93303-93356) and (2) a key word, pattern-based text query of discharge summaries (DS) that selected on the term “endocarditis” in fields headed by “Discharge Diagnosis” or “Admission Diagnosis” or similar. Further coding extracted the nature and type of valve and the organism responsible for the IE if present in DS. All identified cases were chart reviewed using pre-specified criteria for true IE. Positive predictive value (PPV) was calculated as the total number of verified cases over the algorithm-selected cases. Sensitivity was the total number of algorithm-matched cases over a final list of 166 independently identified true IE cases from ID and Cardiology services. Specificity was defined using 119 pre-adjudicated non-cases minus the number of algorithm-matched cases over 119.

**Results:**

The ICD-based query identified 612 individuals from July 2015 to July 2019 who had a hospital billing code for infective endocarditis; of these, 534 also had an echocardiogram. The DS query identified 387 cases. PPV for the DS query was 84.5% (95% confidence interval [CI] 80.6%, 87.8%) compared with 72.4% (95% CI 68.7%, 75.8%) for ICD only and 75.8% (95% CI 72.0%, 79.3%) for ICD + echo queries. Sensitivity was 75.9% for the DS query and 86.8-93.4% for the ICD queries. Specificity was high for all queries >94%. The DS query also yielded valve data (prosthetic, tricuspid, pulmonic, aortic or mitral) in 60% and microbiologic data in 73% of identified cases with an accuracy of 94% and 90% respectively when assessed by chart review.

Table 1. Test Characteristics of Three Electronic Health Record Queries for Infective Endocarditis

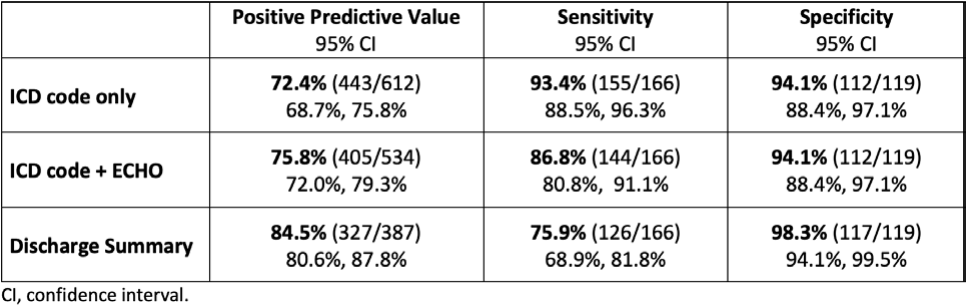

**Conclusion:**

Compared to traditional ICD-based queries, text-based queries of discharge summaries have the potential to improve precision of IE case ascertainment and extract key clinical variables.

**Disclosures:**

**All Authors**: No reported disclosures

